# Ocular transient receptor potential channel function in health and disease

**DOI:** 10.1186/s12886-015-0135-7

**Published:** 2015-12-17

**Authors:** Peter S. Reinach, Stefan Mergler, Yuka Okada, Shizuya Saika

**Affiliations:** Department of Ophthalmology and Optometry, Wenzhou Medical University, 270 Xuejuan Road, Wenzhou, Zhejiang 325027 P. R. China; Department of Ophthalmology, Charité-University Medicine Berlin, Campus Virchow-Clinic, Augustenburger Platz 1, 13353 Berlin, Germany; Department of Ophthalmology, Wakayama Medical University School of Medicine, Wakayama, Japan

**Keywords:** Transient receptor potential ion channels, Calcium, Retina, Cornea, Uvea, Lens

## Abstract

Transient receptor potential (TRP) channels sense and transduce environmental stimuli into Ca^2+^ transients that in turn induce responses essential for cell function and adaptation. These non-selective channels with variable Ca^2+^ selectivity are grouped into seven different subfamilies containing 28 subtypes based on differences in amino acid sequence homology. Many of these subtypes are expressed in the eye on both neuronal and non-neuronal cells where they affect a host of stress-induced regulatory responses essential for normal vision maintenance. This article reviews our current knowledge about the expression, function and regulation of TRPs in different eye tissues. We also describe how under certain conditions TRP activation can induce responses that are maladaptive to ocular function. Furthermore, the possibility of an association between TRP mutations and disease is considered. These findings contribute to evidence suggesting that drug targeting TRP channels may be of therapeutic benefit in a clinical setting. We point out issues that must be more extensively addressed before it will be possible to decide with certainty that this is a realistic endeavor. Another possible upshot of future studies is that disease process progression can be better evaluated by profiling changes in tissue specific functional TRP subtype activity as well as their gene and protein expression.

## TRP channel characteristics

In 1969, a Drosophila mutant was identified having defective vision (abnormal electroretinogram) in which light-induced channel activation elicited a transient rather than sustained membrane voltage depolarization [1–3]. About 20 years later, this aberrant behavior was attributed to a mutant TRP gene. Subsequent to its cloning in 1989 it was realized that a mutation in the *trp* gene underlay this aberrant light response. Specifically, cloning and sequencing of the Drosophila *trp* gene showed that its 4.1-kb RNA transcript encodes a 1275—amino acid protein [1, 4]. Since extracellular application of the non-specific Ca^2+^ channel blocker lanthanum-III-chloride (La^3+^) to the retina of the blowfly *Calliphora* caused extreme weakening of the receptor potential to the dark baseline level during a light pulse [1]. This response showed that *trp* is a light sensitive channel. This breakthrough led to the realization that other channels with unique structures had nevertheless a signature TRP sequence (i.e. TRP box).

These TRP box containing channels are characteristic of a superfamily in mammals derived from 28 different genes. They are further categorized by separating them into six different subfamilies based on their sensitivity to activation by different stimuli. TRP receptor channel activation in different ocular tissues is essential for not only visual processing, but also the maintenance of normal health. These non-selective channels transduce environmental stresses into cell signaling events controlling different adaptive responses needed for offsetting such challenges. Emerging indications of their importance has prompted efforts to identify lead compounds, which can modulate their activation profiles in order to counter losses in cellular function caused by tissue injury, touch, fluctuations in ambient temperature, pH as well as medium osmolarity, hormonal exposure and pathogen infiltration. This endeavor can lead to the development of novel strategies for improving the treatment of different ocular diseases in a clinical setting.

TRPs are non-selective cation channels with variable Ca^2+^permselectivity. Their activation by a vast array of different stimuli elicits intracellular Ca^2^ transients leading to downstream stimulation of various signaling pathways as well as in some cases transactivating other receptor types. It is still unclear how TRP responses encode through modulation of Ca^2+^ influx a myriad of downstream signaling events triggering response formation [5–7].

We review here TRP functional involvement in cornea, conjunctival epithelium, uvea, lens and retina. It is our intent that the reader will a) more fully appreciate their importance in maintaining ocular function; b) realize that changes in TRP functional expression can underlie ocular disease.

## TRP channel channelopathies and nomenclature

TRPs sense diverse environmental stimuli including ambient temperature, changes in membrane stress, declines in pH, exposure to anisosmotic media and environmental ligands as well as kinase-induced channel phosphorylation [8–10]. Changes in their function due to mutation [so-called (TRP) *channelopathies*] are associated with human diseases such as cancer [6, 11–13]. There are strong indications that variant TRP expression is also involved in many diseases (e.g. mutations in TRPs are responsible for various kidney diseases), or cancer (e.g. prostate, breast, colon, rectum) [6, 14]. Structure function TRP relationship studies contribute to development of novel strategies for reversing dysfunctional responses underlying various pathological conditions.

TRPs consist of six putative transmembrane domains with a pore loop between the fifth and sixth spanning segments. The span between the fifth and sixth segments forms a conduit for cation membrane permeation. Each channel is composed of four subunits that can be organized in either a heteromeric or homomeric configuration. These different subunit combination possibilities account for Ca^2+^ selectivity variability. Various different subfamilies of TRPs are known [7, 15–18]. Based on amino acid sequence and functional similarities, TRP channels are divided into seven main subfamilies: a) TRPA (ankyrin); b) TRPC 1-4 (canonical); c) TRPM1-8 (melastatin) [19, 20]. TRPM8 (originally named Trp-p8) is a cold- and menthol-sensing Ca^2+^-permeable channel which plays a crucial role in cold thermosensation; Except for TRPM4 and TRPM5, all other TRPs are permeable to Ca^2+^ [12, 21]. d) TRPML (mucolipin); e) TRPN (no mechano potential); f) TRPP (polycystin: g) TRPV1-7 (vanilloid) [5, 22]. Drosophila phototransduction is mediated by a TRPC channel together with a second TRPC channel trp-like (TRPL) [23, 24].

## Impact of altered TRP expression on cellular functions and disease states

Modulation of TRP channel activity contributes to the control of cell growth, differentiation, proliferation or secretion through changes in intracellular Ca^2+^ levels. This can trigger downstream linked signaling pathways controlling gene expression events underlying each of these responses. On the other hand, aberrant enhanced TRP channel activation leading to non-physiological rises in extracellular calcium influx or intracellular calcium release from stores (e.g. endoplasmic reticulum) by Ca^2+^-release channels (e.g. IP_3_, ryanodine receptors) can induce apoptosis in various cell types. For example, Yoon et al. found that there is an extremely rapid photoreceptor cell death when a TRP channel is constitutively active due to mutations [25]. In the corneal endothelium, changes in TRP channel activity affect its role in maintaining tissue transparency (barrier and pump function) [26]. On the other hand, calcium overload caused by growth factor-induced TRP channel activation can lead to calcium overload and excessive apoptosis [27, 28]. Such a change can as well compromise organ transplantation success and pathogenesis of eye diseases (e.g. corneal dystrophy) and may contribute to donor cornea shortage [29].

### Determinants of TRP thermosensitivity

Out of the 28 mammalian TRP genes, 6 different thermosensitive TRP isotypes are expressed in the eye. They include TRPV1, 2, 3 and 4 as well as TRPM8 and TRPA1 whose temperature sensitivities cover most of the environmental conditions that mammals encounter. TRPV4 and TRPV3 are activated by temperatures from 25 to 31 °C, respectively, whereas TRPV1 is activated at 43 °C and TRPV2 at a noxious temperature of 51 °C [30]. TRPM8 and TRPA1 on the other hand sense cooling once the temperature drops below 25 and 17 °C, respectively. It is unclear whether one of these three suggested mechanisms can account for the origin of the remarkably steep temperature sensitivity of the thermosensitive TRPs (thermo-TRPs). They include: 1) steep specific temperature dependence for ligand binding; 2) temperature-induced channel rearrangement; 3) temperature-dependent membrane tension changes controlling channel opening [31]. A definitive explanation for why only certain TRPs are thermosensitive remains somewhat elusive. Nevertheless, a recent report suggests that there are specific TRP channel molecular determinants endowing thermal sensitivity. In Drosophila, different TRPA1 isoforms are expressed and the requirement for TRPA1 thermal sensitivity was linked to the expression of a specific 37-amino-acid sequence within its intracellular region (encoded by a single exon) [32].

Cataloging which TRP subtypes elicit specific responses can be problematic if the characterization is based solely on drug-induced effects. This limitation stems from the fact that their effects are not restricted to interacting with a single subtype. Accordingly, transgenic animals are used to assess the impact of either a gain or loss of TRP function on an induced response. Even such a precaution may be confounded by compensatory upregulation of another gene replacing the functional loss resulting from a deleted gene. Despite these possible caveats, current assignments of functional roles of TRP channel subtypes appear to be accurate.

### Classical activation mechanism

Initially, it was realized that either exogenous small organic synthetic compounds or natural products can activate TRPs. Besides capsaicin, [33] other (synthetic) agonists are icilin (TRPM8) [34] and camphor (TRPV3) [35, 36] which are super cooling agents. Furthermore, endogenous lipids or products of lipid metabolism are also ligands of TRPs. For example, anandamide is an endogenous lipid [37]. Inorganic ions such as Ca^2+^ or Mg^2+^ can also (directly) activate TRPs (e.g. TRPM6 for Mg^2+^) [38] (e.g. TRPA1 for Ca^2+^) [39]. However, it is not yet known if the TRP conformational changes induced by Ca^2+^ or Mg^2+^ are the same as those induced by a thermal transition known to activate these different TRP channel subtypes. Overall, classical activation of TRPs and in particular thermo-TRPs are mostly related to direct activation via mechanical stimuli, channel phosphorylation, certain exogenous and endogenous agents or inorganic ions.

### TRP channel regulation by G protein-coupled receptors

Studies in Drosophila photoreceptors by Devary et al. demonstrated for the first time that the light activated TRP channels and TRPL are targets of G protein-coupled receptor (GPCR) activated rhodopsin, which lead to activation of Gq, PLC and PIP_2_ hydrolysis [40]. Another study by Hardie et al. showed for the first time that the DAG branch of PI-signaling (and PUFAs) also activates in Drosophila TRP and TRPL channels [23]. Therefore, TRPs can also be (indirectly) activated by GPCRs and receptor tyrosine kinases (RTKs) activating phospholipase C (PLC). This can occur in different ways such as generation of inositol (1,4,5) trisphosphate (IP_3_) and subsequent Ca^2+^ release from intracellular stores, which in turn activates store-operated Ca^2+^ channels (SOCs) composed of TRP subunits such as TRPC1 and TRPC4 [41]. Another way which GPCRs can modulate TRP channel activity is via the generation of diacylglycerol (DAG) (e.g. diacylglycerol-sensitive TRPC3/6/7) [42]. Finally, GPCRs can modulate TRPs via hydrolysis of phosphatidylinositol (4,5) bisphosphate (PIP_2_) (TRPM8) [43] (TRPC4) [44].

## TRPs in ocular tissues and cells

### Uvea, retina and retinal pigment epithelium

Besides the TRP channel subtypes mediating retinal phototransduction in the Drosophila eye [1, 2, 45, 46], other subtypes identified in the mouse retina include: TRPC1-4 TRPM1/3/7, TRPML1, TRPP2, TRPV2, TRPV4 [47, 48]. In mouse retinal ganglion cells (RGCs), TRPV4 modulates calcium flux, spiking rate, and apoptosis of these cells [48]. TRPV1 in rat retina contributes to eliciting RGC apoptosis and increased intracellular Ca^2+^ levels during exposure to elevated hydrostatic pressure [49]. Furthermore, TRPC1 and TRPC4 are expressed in chicken retina [50]. In the human retina, TRPM1 expression was detected on ON-bipolar cell dendrites. This suggests a dual function for TRPM1 in the ON-pathway [51]. More specifically, TRPM1 is required for the photoresponse in mouse retinal ON-bipolar cells and it is regulated by the metabotropic glutamate receptor 6 (mGulR6) cascade in ON-bipolar cells [52–56]. Many human mutations affected this system [53]. Other TRPs like TRPV1, TRPM8 and TRPA1 are expressed in retinal tumor cells (retinoblastoma). On the other hand, TRPA1 is completely suppressed in a retinoblastoma cell line, which is resistant to the cytostatic agent etoposide [57]. In addition, TRPV1-4, TRPM8 and TRPA1 were identified in retinal pigment epithelial (RPE) cells [58, 59]. Increasing the ambient temperature or insulin like growth factor-1 (IGF-1) enhanced vascular endothelial growth factor-A (VEGF-A) secretion rate in RPE cells [58]. TRPs are involved in the IGF-1 induced response [58]. Regarding the human uvea, there is only one study demonstrating gene expression of TRPV1, TRPM8 and TRPA1 [59]. In contrast, in human uveal melanoma cells, the gene expression of TRPM8 is at lower levels whereas the TRPA1 expression is at high levels in healthy uvea [59].

### Corneal epithelium

TRPC4 is the first TRP channel subtype identified in human corneal epithelial cells (HCEC). Activation by epidermal growth factor of its cognate receptor, EGFR, transactivates TRPC4. The resulting increases in intracellular trigger mitogen activated protein kinase (MAPK) cascade signaling leading to rises in cell proliferation and migration in vitro and in vivo [60]. Conversely, TRPV1 activation by capsaicin, induces increases in cell proliferation and migration through mediating increases in release of heparin bound EGF, which transactivates EGFR [61]. TRPV1-4 vanilloid are expressed in rat, mice and HCEC [62–64]. Thermal transitions were used to delineate functional TRPV1-4 [65–72] and TRPM8/TRPA1 [73, 74] expression.

TRPV1 expression in HCEC has potential clinical relevance since in TRPV1 knockout mice re-epithelialization and restoration of tissue transparency following debridement was delayed in these mice compared to their wild type counterpart. This effect of TRPV1 in promoting cell proliferation and migration is associated with increases in IL-6 and substance P expression, which are coactivators of growth factor induced wound healing [62]. TRPV4 expression in intact human corneal epithelium and its activation by exposure to a hypotonic challenge is required for inducing regulatory cell volume decrease behavior [75]. Furthermore, Pan et al. [76] found that hypertonic stresses identical to those described in some dry eye patient tears elicited TRPV1 channel stimulation leading to rises in proinflammatory cytokine levels through MAPK and NF-kB activation [64, 77]. TRPM8 cold receptor gene, protein and functional expression was detected in HCEC. On the other hand, TRPM8 activation by either temperature lowering or icilin inhibits TRPV1-induced increases in intracellular Ca^2+^ levels (Türker, Mergler et al. unpublished observation 2015).

### Corneal stroma

Functional TRPV1 expression was identified in human stromal fibroblast cultures [78, 79]. Transforming growth factor TGFβ-1 transactivates TRPV1 in these cultures through activation of its cognate receptor, TGFβR. In vivo, a murine corneal alkali burn compromises basement membrane integrity leading to TGFβ stromal infiltration and myofibroblast transdifferentiation, fibrosis and a proinflammatory cytokine, chemoattractant storm followed by immune cell infiltration. These injury-induced effects cause corneal opacification and ulceration [80]. This marked difference in the wound healing outcome resulting from epithelial and stromal TRPV1 activation suggests that TRPV1 antagonist usage in a clinical setting may need to be restricted to cases involving penetrating stromal injury rather than superficial epithelial injury. TRPM8 gene, protein and functional expression was validated in human corneal stromal cells (Türker, Mergler et al. unpublished observation 2015).

### Corneal nerve fibers

Thermosensitive TRPA1 and TRPM8 are also expressed on corneal afferent nerves [81]. Corneal nerve fibers are radially distributed within the stroma and the adjacent limbus [82]. TRPV1 and TRPM8 are highly expressed in mouse, guinea pig and human corneal sensory nerve fibers at levels similar to those in their non-corneal counterpart [81, 83–85] and in non-corneal primary sensory neurons [30]. These channels desensitize during prolonged exposure to capsaicin (TRPV1) or menthol (TRPM8). In a clinical setting, nerve fiber TRPM8 activation by a super cooling agent may provide an option for treating dry eye syndrome since corneal cooling in humans increased lacrimation, whereas warming had an opposite effect [81]. Parra et al. found that TRPM8 functional expression is needed to maintain ocular surface hydration during exposure to a desiccating stress [81]. Taken together, these results suggest that drug-induced fibroblast TRPV1 and neuronal TRPM8 functional modulation may provide novel options to lessen corneal opacification and increase lacrimation subsequent to a penetrating corneal injury and in aqueous deficient dry eye disease, respectively.

### Corneal endothelium

Rae and Watsky [86] described in the corneal endothelium an outwardly rectifying thermosensitive current along with a K^+^-selective current. The origin of the thermosensitive component was not identified, but may be attributable to TRP activity [86]. More recently, thermosensitive TRPV1-4 channel activity was identified in human corneal endothelial cells [87, 88]. Besides voltage-operated Ca^2+^ channels (VOCCs) [27, 28], there is functional menthol receptor TRPM8 expression in human corneal endothelial cells because the super cooling TRPM8 agonist icilin [34] reversibly increased intracellular Ca^2+^levels [89]. Besides TRPM8 expression in human corneal endothelial cells, Mergler et al. [28] suggested that other TRP subtypes may be also expressed in human corneal endothelium since H_2_O_2_ induced significant rises in [Ca^2+^]_I_ levels. This Ca^2+^ rise could be due to activation of redox sensitive TRP subtypes mediating previously unexplained biological phenomena and are involved in various pathologies [90]. Corneal endothelial functional TRPM8 expression may help explain why storing isolated corneas in an eye bank setting at temperatures below those in-situ results in less swelling than at 37 °C [89, 91, 92]. TRPM8 stimulation could result in graded shifts of voltage-dependent activation to more negative membrane voltages increasing the electrical driving force for intracellular Ca^2+^ influx [31]. TRPM8 may be also regulated by G protein-coupled receptors (GPCRs) since both activation of a GPCR and a nerve growth factor receptor inhibited menthol- and cold-induced TRPM8 activity in non-corneal cells [20].

### Lens epithelium

Intracellular Ca^2+^ regulation in lens epithelial and fiber cells is critical to lens transparency maintenance since rises in its level are cataractogenic. Functional TRPM3 and TRPV1 expression was identified in human lenses [93]. TRPM3 variants are associated with cataractogenesis [93]. TRPV4 expression in porcine lens epithelium regulates hemi-channel-mediated ATP release and Na^+^- K^+^-ATPase activity [94]. Plasma membrane associated store operated channel (SOC) activity is modulated through changes in the intracellular store (ICS) Ca^2+^ filling status. Such feedback control of SOC activity is mediated by TRPC1 and TRPC5 subtypes whose open time is prolonged following ICS Ca^2+^ depletion [76].

### Conjuncitval epithelial cells

In conjunctival epithelial cells, there are temperature-sensitive TRPV1, TRPV2 as well as TRPV4 channels [95]. There are indications for crosstalk between TRPV1 and TRPM8 since TRPM8 activation suppressed TRPV1-induced Ca^2+^ increases as well as increases in proinflammatory cytokine IL-6 release [96].

## Adaptive roles of functional TRP expression

TRPs make an important contribution to maintaining tissue homeostasis under a variety of environmental conditions that can be otherwise disruptive. In humans, TRPs play an important role in modulating taste sensation, eliciting responses to painful stimuli, temperature, growth factors and pheromones [97–103]. Modulation of their activity and expression can also change the effects of stressors mediating cell death [49, 102, 104]. In addition, store-operated Ca^2+^ channels (SOCs) can be activated via intracellular store (ICS) calcium depletion and as aforementioned by activated GPCRs or receptor-linked tyrosine kinases (RTKs). However, their dysfunction is linked with sustained increases in cytosolic Ca^2+^ leading to apoptosis [97–99, 101, 105].

### Healthy eyes

Worldwide, the number of people needing relief from ocular dysfunction is on the rise. One of the reasons for this increase is a change in lifestyle due to reliance on video display terminals and stresses imposed by urbanization. Corneal thermosensitive TRPV1-4, TRPA1 and TRPM8 channels in the cornea can be activated by stresses encountered in daily living and elicit responses that can contribute to ocular disturbances. In addition, 1) TRPV1 (capsaicin); 2) TRPV2/3 (camphor, laurel tree *cinnamomum camphora*)*; 3)* TRPV4, bisandrographolide in Chinese herbal plant *Andrographis paniculata; 4)* TRPM8, menthol in green mint; 5) TRPA1, cinnamaldehyde, isothiocyanates and allicin in horseradish are selectively ligand gated [106]. An indication of the adaptive value of TRPV1 expression in the cornea is that application of capsaicin to the ocular surface elicits excruciating pain, which elicits an avoidance response to reduce tissue injury. Thermosensitive corneal TRP expression may also provide an adaptive advantage by eliciting responses that reduce disruptive noxious thermal effects on tissue homeostasis. Similar considerations are relevant to the posterior section of the eye such as retina, uvea and retinal epithelium (RPE) since in these tissues there is also thermosensitive TRP expression (e.g. TRPV2 in RPE) [58]. On the other hand, retinal TRPV1 expression level is low compared to that in the anterior section of the eye (e.g. lens) [107]. Furthermore, the menthol receptor, TRPM8, provides essential functions including contributing to controlling basal tear fluid secretion via corneal nerve fibers [81, 83]. In addition, human corneal endothelial TRPM8 expression may provide an adaptive advantage for preserving tissue function during temperature lowering [89]. In summary, functional thermosensitive TRP activation by environmental challenges elicits essential responses that have adaptive value in preventing or reducing tissue function compromise.

### Eye diseases

With the realization in the last decade of the polymodal regulatory roles of TRP channels, there is heightening interest in studying how they elicit such control. This is evident based on the ever-increasing number of reports appearing in Pub Med and their citation frequency. One of the realizations sparking such interest is that TRP gene mutations underlie numerous inherited diseases in humans including the eye [108]. TRP channelopathies were initially linked to cardiovascular, renal, skeletal and nervous system pathology [108–110]. Regarding the eye, one of them deals with mucolipidosis type IV (MLIV) and another indicates that mutant TRPM3 expression is associated with cataractogenesis and glaucoma [93]. MLIV is an autosomal recessive, neurodegenerative lysosomal storage disorder, which is due to mutations in the gene MCOLN1. MLIV is clinically characterized not only by ophthalmologic abnormalities such as corneal opacity, retinal degeneration and strabismus, but also by other non-ocular abnormalities. MCOLN1 which encodes the protein mucolipin 1 (MNL1) is a non-specific cation channel (TRPML1) (review [111]). In addition, human TRPM1 mutations are associated with congenital stationary night blindness (CSNB), whose patients lack rod function and suffer from night blindness starting in early childhood [112].

#### Dry eye syndrome

**Fig. 1:**
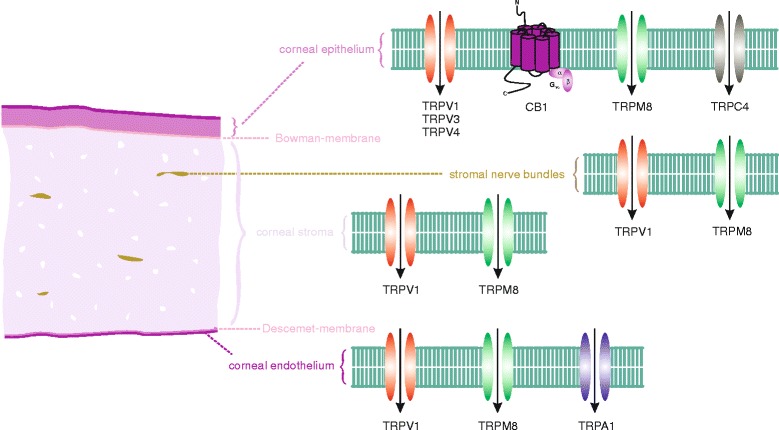
TRP channels and cannabinoid receptor 1 (CB1) in the human cornea: Corneal epithelium: TRPV1/3/4 [64, 75, 140], CB1 [141], TRPC4 [60], TRPM8 (Khajavi, Mergler et al., 2014, unpublished observation); corneal stroma: TRPV1 [78], TRPM8 (Türker, Mergler et al., 2015, unpublished observation); corneal nerve fibers: TRPV1 [142], TRPM8 [81]; corneal endothelium: TRPV1-3 [88], TRPV4 [87]; TRPM8 [89], TRPA1 [89]

The options for treating dry eye syndrome (DES) are limited for the most part to providing palliative relief. With the identification of anterior segment functional TRP expression illustrated in Fig. 1, there is suggestive evidence that they are potential drug targets for improving treatment of this disease. DES or keratoconjunctivitis sicca is a complex multifactorial disease characterized by an immune and inflammatory process that affects the lacrimal glands and ocular surface [113]. TRPV1 is implicated as a possible drug target since TRPV1 activation hastens corneal epithelial wound healing whereas its inhibition reduces stromal opacification and hypertonic-induced inflammation [80, 114, 115]. TRPM8 activation on afferent sensory corneal nerves and in the different corneal cell types enhances basal tear flow during a decline in ambient temperature [81, 95, 115].

#### Diabetic retinopathy

Ocular diseases such as diabetic retinopathy can be a manifestation of systemic diabetes mellitus. One of its pathophysiological alterations is impaired retinal blood flow due to vascular occlusion. This condition can cause photoreceptor and neuronal hypoxia leading to cell death and visual impairment. Whereas there are various studies describing a connection between TRPs and diabetes mellitus (review [116, 117]), there are no studies directly related to diabetic retinopathy. On the other hand, some are available pertaining to non-diabetic related retinopathy (e.g. melanoma-associated retinopathy) [112, 118, 119].

#### Glaucoma

In glaucoma, one of its pathological effects can includes declines in retinal ganglion cell (RGC) function due to intraocular pressure elevations compressing nerve fibers traversing through the optic nerve head. Changes in TRP function may contribute to changes in RGC function induced by such stress [49, 120]. Other tissue changes that may contribute to glaucoma damage are obstruction of aqueous outflow pathways through the trabecular meshwork and/or a change in ciliary muscle contractility. As some mechanosensitive TRPC channel subtypes are expressed in these tissues [121], a change in their store-operated channel activity could contribute to trabecular meshwork (TM) pathology [76]. Functional mechanosensitive TRPV4 expression on primary cilia in TM cells transduces pressure changes resulting from variations in aqueous humor formation. This subtype is implicated in how the eye senses such variations to maintain normostensive aqueous humor outflow Its regulatory role suggests that TRPV4 is an attractive therapeutic target for the treatment of hypertensive glaucoma [121]. In another study, TRPC6 gene expression in primary open-angle glaucoma patient leukocytes was detected. Interestingly, its expression was higher than that in control cataract patients, which suggests this channel subtype is a relevant biomarker for this kind of eye disease [122]. Additionally, changes in TRPC6 gene expression level were also correlated with alterations of intraocular pressure and cup-to-disc ratio. However, treatment with different anti-glaucoma drugs did not affect its gene expression. This negative effect illustrates the complexities involved in understanding the pathophysiology of glaucoma.

### Ocular tumors

Ocular tumor development is a rare occurrence compared to its prevalence in non-ocular tissues. In choroidal- or corneal neovascularization, this process is not directly related to tumor neovascularization. Nevertheless, neovascularization is a dangerous process whose progression determines tumor growth and metastasis. Endothelial cells (ECs) play a role in neovascularization and several different TRP subtypes have been detected in this tissue (review [123]). At this point, Ca^2+^ channel drug targeting is being considered in non-ocular tissues (review [124, 125]) and ocular tissues, but findings in these studies are unrelated to tumor neovascularization. One study showed that blockage of Ca^2+^ activated K^+^ channels inhibited angiogenesis induced by epidermal growth factor (EGF) [126]. In retinal pigment epithelium (RPE), activation of L-type Ca^2+^ channels is associated with increases in vascular endothelial growth factor (VEGF) secretion. Additionally, basic fibroblast growth factor (bFGF) increased L-type channel activity [127, 128]. A follow-up study demonstrated in the RPE that TRPV2 channels mediate increases in both heat-dependent and IGF-1 (via PI3-kinase activation)-induced VEGF secretion through rises in intracellular Ca^2+^ levels.

#### Uveal melanoma

Uveal melanoma (UM) is a devastating disease in which patient survival rates are poor once this tumor metastasizes out of the eye into the liver, lung, bone and skin. It is the second most prevalent malignant tumor of melanocytes [129, 130]. This disease directly develops from degenerated melanocytes in the choroid. TRPs expression was identified in healthy non-ocular and ocular melanocytes [131]. Specifically, TRPM1 was designated as a melanoma metastasis suppressor based on its expression in normal pigment cells in the skin and its absence in aggressive ocular metastatic -competent melanomas. A similar association pertains to TRPA1 because its expression is lower in human uveal melanoma cells than in healthy uvea [59]. An inverse relationship was described for TRPM8 which is expressed at higher levels in most of the investigated UM cell lines [59]. Furthermore, TRPV1 and the cannabinoid receptor 1 (CB1) are functionally expressed in UM cells. Interestingly, activation of CB1 induced Ca^2+^ transients, which were suppressed by either La^3+,^ a non-selective TRP channel blocker and capsazepine, a selective TRPV1 antagonist. On the other hand, capsaicin-induced Ca^2+^ transients could also be suppressed by CB1 activation. Therefore, it is suggested that identification of functional TRPV1, TRPM8, TRPA1 and CB1 expression in uveal melanoma may provide novel drug targets for treatment of this aggressive neoplastic disease [59]. A similar suggestion was made for treating non-uveal melanoma (TRPM8) [132].

#### Retinoblastoma

**Fig. 2:**
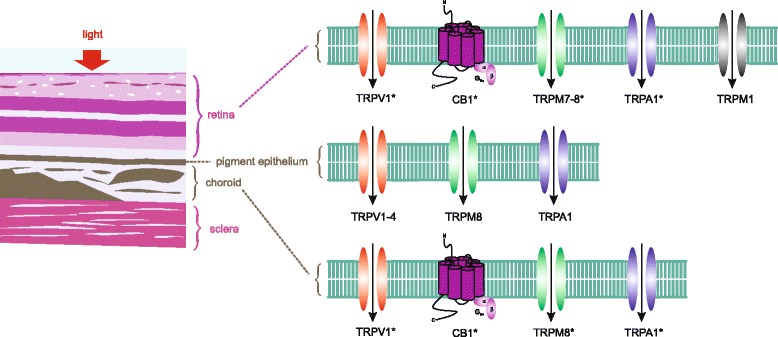
TRP channels and CB1 in the posterior section of the eye: Retina: TRPV1 [57], CB1 [57], TRPM7 [139], TRPM8 [57], TRPA1 [57], TRPM1 [51]. TRPs marked with an asterisk were detected in retinoblastoma cells. Interestingly, TRPA1 could not be detected in etoposide-resistant retinoblastoma cells [57]; retinal pigment epithelium: TRPV1 [58, 59], TRPV2-4 [58], TRPM8 [58], TRPMA1 [58]; choroid: TRPV1 and CB1 [58], TRPM8 and TRPA1 [58], TRPs marked with an asterisk were also detected in uveal melanoma cells. Notably, the gene expression of TRPM8 is at lower levels in uveal melanoma cells whereas the TRPA1 expression is at higher levels in healthy uvea [58]

Figure 2 provides an illustrative representation of the different TRP subtypes expressed in the retina. Retinoblastoma (RB) is a malignant retinal tumor, which develops from immature retinal cells. Its incidence is low, but is the most common ocular tumor of the eye in children and is associated with a RB mutation [133]. There are several established cell lines that serve as retinoblastoma cell models exhibiting voltage-dependent ion channel activity [134–138]. Specifically, the Ca^2+^ channel antagonist mibefradil inhibited cell proliferation via different cytotoxic pathways mediated by voltage-dependent T-type Ca^2+^ channels [136]. Regarding Ca^2+^ permeable TRPs, Hanano et al. suggested a possible significant regulatory role of TRPM7 for retinoblastoma cell proliferation as a spontaneously activated Ca^2+^ influx pathway [139]. Another study revealed TRPV1, TRPM8 and TRPA1 gene expression in retinoblastmoa cells [57]. Notably, the expression of TRPA1 was suppressed in etoposide-resistant RB cells. Therefore, using genetic approaches to upregulate TRPA1 expression could provide a means to induce etoposide sensitivity and suppress RB cell tumorigenesis. Furthermore, CB1 was detected in uveal melanoma cells. Activation of CB1 suppressed TRPV1-induced Ca^2+^ increases in etoposide-sensitive RB cells whereas this effect did not occur in etoposide-resistant RB cells [57]. There have been only a few reports describing a TRP involvement whereas other types of Ca^2+^ channels have been studied more extensively. Nevertheless, the limited TRP characterizations suggest that there are clear differences between healthy and tumorous ocular tissues or cytostatic-resistant ocular tissue. Therefore, modulation of TRP expression and/or function could provide a badly needed therapeutic option. In addition, monitoring TRP expression levels could provide a prognostic marker for identifying this insidious disease.

## Conclusion

Ocular TRP functional expression is essential for mediating both adaptive and maladaptive responses to a wide variety of environmental stressors challenging tissue homeostasis. Table 1 lists ocular tissue TRP subtype localization and pharmacology described in pertinent references. It is still uncertain how the activation of a single TRP subtype by diverse stimuli can mediate a number of different signaling pathways and diverse responses. One tenable explanation is that TRP subtypes can have specific molecular determinants with which activators interact. In other words, it still needs to be understood whether there are specific determinants on a TRP subtype that induce each of the different responses attributable to its activation. Each of these different sites may be linked to diverse signaling pathways mediating variety of different responses. Verification of this possibility requires generating site specific TRP mutants to determine if such alterations correspond with any of the pathophysiological responses by cells expressing TRPs. One example of such an approach would entail delineating why capsazepine interaction with TRPV1 in one setting suppresses injury-induced inflammation, but in another setting it instead elevates body temperature. Nevertheless, based on the burgeoning number of TRP-related publications and commitments pharmaceutical companies are making to identify novel lead compounds for future drug development, it is conceivable that ocular disease management may benefit from such endeavors. This appears to be tenable since there are indications that there is an association between aberrant TRP expression and disease. In any case, much effort must still be committed to determine if there is a cause and effect relationship between specific TRP malfunctions and a pathophysiological condition underlying a eye disease.

**Table 1:** Characterization of TRP channel tissue localization

Name	Selectivity P_Ca_:P_Na_ [12]	Activation threshold temperature (°C) [30]	Pharmacology [5, 42, 143]	Function [5, 30]	Posterior eye section	Anterior eye section
TRPC1	Non-selective	---	Store depletion, 2-APB	Component of SOC	Mouse retina [47, 48]	HCEC [60], TM [121]
TRPC2	2.7	---	DAG	?	Mouse retina [47, 48]	TM [121]
TRPC3	1.6	---	Store depletion, OAG, 2-APB, DAG, Pyr3	Component of SOC	Mouse retina [47, 48]	HCEC [60], TM [121]
TRPC4	1.1–9.0	---	Store depletion 2-APB	Component of SOC	Mouse retina [47, 48]	HCEC [60]
TRPV1	4–10	>43	Capsaicin, capsazepine, anandamide, NADA	Heat sensor osmosensor^a^	Rat retina [49], human retina (tumor) [57], uvea (tumor) [59], human RPE [58, 59]	HCEC [63, 64], HCK [78], HCEC-12 [88]
TRPV2	1–3	>52	Cannabidiol, 2-APB	Heat sensor	Human RPE [58]	HCEC-12 [88]
TRPV3	2,6	30–39	Camphor, 2-APB	Moderate heat sensor	Human RPE [58]	HCEC [144], HCEC-12 [88]
TRPV4	6–10	24–27	4α-PDD, GSK 1016790A	Moderate heat sensor osmosensor^b^	Mouse RGC [48], human RPE [58]	HCEC [75], HCEC-12 [87], HLE [94]
TRPM1	Not determed	---	mGluR6	Transduction of light signals	Mouse retina [47, 48, 145], human retina [51]	---
TRPM2	0.5–1.6		H_2_O_2_, ADP-ribose, β-NAD^+^	Mechanotransduction	---	HCEC-12 [28], TM [121]
TRPM7	3	---	Spermine		Mouse retina [47, 48], human retina [139]	---
TRPM8	1–3.3	<23–28	Menthol, icilin, eucalyptol, BCTC	Moderate cold sensor	Human retina (tumor) [57], uvea (tumor) [59]	HCEC^c^HCNF [81], HCK^c^, HCEC-12 [28, 89]
TRPA1	0,8	<17	Icilin, alicin	Cold sensor	Human retina (tumor) [57], uvea [59]	HCEC-12 [28, 89], TM [121]

*HCEC* human corneal epithelium

*HCK* human corneal keratocytes (stroma)

*HCEC-12* human corneal endothelium

*TM* trabecular meshwork

*HCNF* human corneal nerve fibers

*HLE* human lens epithelium

*RPE* retinal pigment epithelium

*RGC* retinal ganglion cells

^a^activation by hypertonic challenge

^b^activation by hypotonic challenge

^c^Mergler et al. 2015 (unpublished data)

## Abbreviations

TRPs: Transient receptor potential ion channels
[Ca^2+^]_i_: Intracellular Ca^2+^ concentration
